# A surprising complete radiographic response to palbociclib plus endocrine therapy in an HR-positive, HER2-low lung, pleural, and lymph node metastatic breast cancer patient: a case report

**DOI:** 10.3389/fonc.2026.1856673

**Published:** 2026-10-07

**Authors:** Mingzhi Cha, Hao Yi, HuiMing Li, Rongrong Peng

**Affiliations:** 1 Department of Breast Surgery, Pingxiang People’s Hospital, Pingxiang, Jiangxi, China; 2 Department of Pathology, Pingxiang People’s Hospital, Pingxiang, Jiangxi, China

**Keywords:** breast cancer, CDK4/6 inhibitor, palbociclib, prognosis, targeted therapy

## Abstract

Hormone receptor-positive, human epidermal growth factor receptor 2-low (HER2-low) metastatic breast cancer (MBC) constitutes the predominant subtype of advanced breast cancer, with most patients eventually developing resistance to endocrine therapy (ET) and cyclin-dependent kinase 4/6 inhibitors (CDK4/6i). This case report describes a 64-year-old woman with hormone receptor (HR)-positive, HER2-low MBC involving lung, pleural, and lymph node metastases who achieved a remarkable and sustained radiographic complete response (rCR) to palbociclib plus fulvestrant, followed by letrozole after fulvestrant injection intolerance. The patient exhibited near-complete regression of metastatic lesions within 18 months and maintained remission for over 6 years, far exceeding the typical median progression-free survival observed in clinical trials. The prolonged remission observed underscores the existence of biologically distinct tumor subsets with exceptional sensitivity to CDK4/6 inhibition, warranting investigation into underlying molecular mechanisms.

## Introduction

Hormone receptor-positive, human epidermal growth factor receptor 2-negative (HER2-negative) metastatic breast cancer (MBC) represents the most common subtype of advanced breast cancer, accounting for approximately 70% of cases (1, 2).

Clinical investigations including PALOMA-2, Monaleesa-2, and Monarch-3 have established the therapeutic potential of cyclin-dependent kinase 4/6 (CDK4/6) inhibitors (palbociclib, ribociclib, and abemaciclib) when administered alongside endocrine therapy (ET). These combination regimens have shown particular efficacy in improving progression-free survival (PFS) and overall survival (OS) outcomes in first-line treatment settings (3–5). Based on these robust clinical findings, major oncology guidelines from the National Comprehensive Cancer Network (NCCN), European Society for Medical Oncology (ESMO), and Chinese Society of Clinical Oncology (CSCO) now recommend CDK4/6 inhibitor–ET combinations as the primary treatment approach for hormone receptor (HR)-positive, HER2-negative metastatic breast cancer (6–8). Although the optimal sequencing of CDK4/6 inhibitors and endocrine therapy continues to be discussed, current international guidelines still recommend CDK4/6 inhibitor-based endocrine therapy as the standard first-line treatment for most patients with HR-positive, HER2-negative metastatic breast cancer (9). The phase III SONIA trial found that first-line use of CDK4/6i + ET did not demonstrate a statistically significant PFS benefit for first-line versus second-line use of a CDK4/6 inhibitor plus ET, and first-line use may increase toxicity and cost. These findings underscore the need to further define the optimal sequencing of CDK4/6 inhibitors in patients with HR-positive, HER2-negative advanced breast cancer (9).

The combination of CDK4/6i with ET has become the first-line standard for HR-positive, HER2-negative MBC, demonstrating significant improvements in PFS and OS (10). However, complete responses remain rare, and durable remissions beyond 5 years are exceptionally uncommon, particularly in patients with visceral metastases such as pleural involvement (11). HER2-low tumors have emerged as a clinically relevant subgroup, particularly in the era of antibody–drug conjugates, although their predictive value for response to CDK4/6 inhibitor-based therapy remains uncertain (12, 13).

We report a patient with HR-positive, HER2-low metastatic breast cancer involving the lung, pleura, and lymph nodes who achieved a significant and sustained radiographic complete response with palbociclib plus fulvestrant followed by palbociclib plus letrozole. After 18 months of treatment, the patient’s metastatic lesions were nearly completely resolved. She switched ET drug type because of injection intolerance after 24 months of treatment, but the remission still lasted for more than 6 years and continues to this day. This case highlights the significant potential of palbociclib in combination with ET in selected patients with HR-positive, HER2-low advanced breast cancer and may inform treatment decisions in similar patients. However, the underlying mechanism needs to be further studied.

## Case presentation

### Demographic information and clinical findings

A 64-year-old untreated postmenopausal woman was hospitalized with “cough, hoarseness for more than half a month, and a 1-day discovery of a mass in the left breast.” No genetic, family, or psychosocial history was reported. Physical examination showed that a 6.0 * 5.0 cm area of glands was palpable from the upper outer quadrant of the left breast to the nipple area, which was thickened and hardened, adhered to the nipple, and had no abnormal secretion.

### Diagnostic assessments

An imaging examination from the initial diagnosis is shown in **Figure 1** (2019.11.22).

**Figure 1:**
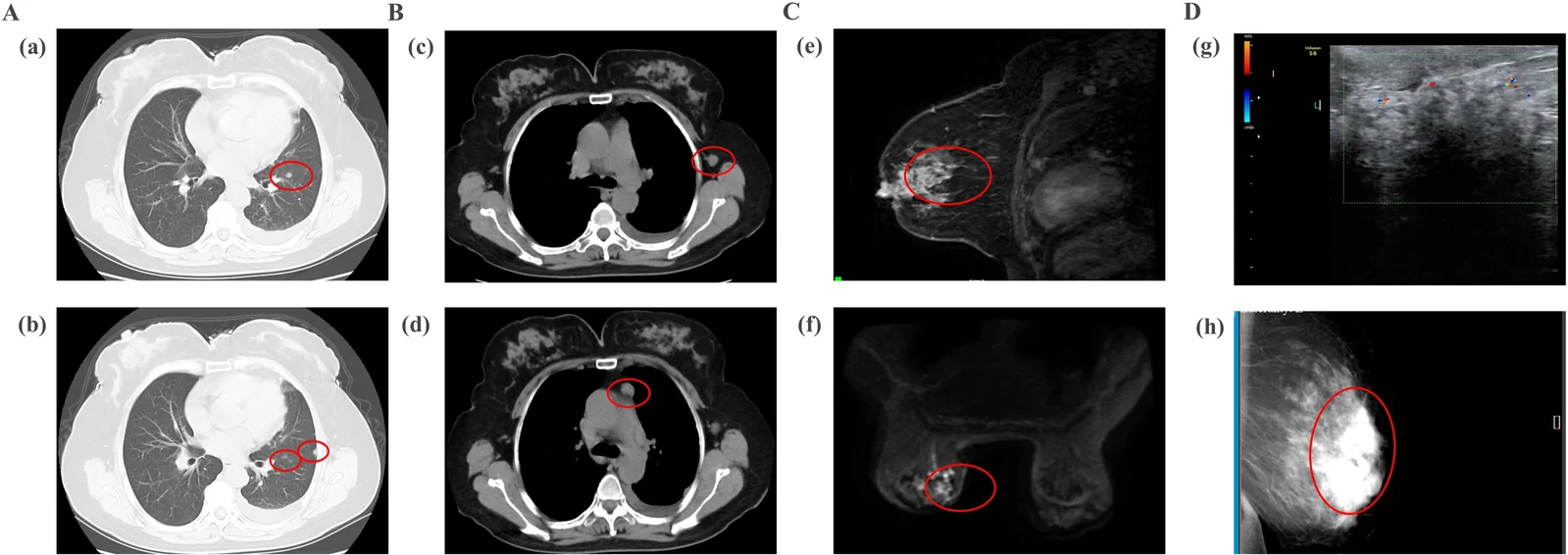
Imaging examinations of the patient at the initial diagnosis. **(a)** CT revealed metastatic nodules in the lung. **(b)** CT revealed metastatic nodules in the pleura and pleural effusion. **(c)** Computed tomography revealed axillary lymph node metastasis. **(d)** CT revealed mediastinal lymph node metastasis. **(e, f)** Breast MRI showed signs of left breast malignancy. **(g)** Breast color Doppler ultrasound showed signs of malignant tumor in the left breast. **(h)** Mammography showed signs of malignant tumor in the left breast.

#### Hematological tumor markers

The results were as follows: carcinoembryonic antigen (CEA) (0–35 U/mL) 2.58 U/mL, Carbohydrate Antigen 15-3 (CA153)(CA153 (0–25 U/mL) 27.61 U/mL ↑, CA199 (0–27 U/mL) 7.44 U/mL, CA125 (0–35 U/mL) 154.1 U/mL ↑, and CA211 (0–3.3 ng/mL) 4.48 ng/mL ↑. The changes and records of tumor markers in the subsequent follow-up are available in **Table 1**.

**Table 1:** Follow-up records of blood tumor marker detection.

Examination time	2019.11.22	2020.2.26	2020.7.27	2020.9.16	2021.1.26	2021.5.19	2022.2.12	2022.8.2	2024.8.21	2024.10.17	2024.10.17	2025.5.20	2025.12.17
CEA (0–35 U/mL)	2.58	1.44	3.2	2.52	1.94	1.51	2.9	3.36	2.26	1.99	1.99	1.6	1.63
CA153 (0–25 U/mL)	↑ 27.61	↑ 32.14	13.47	14.07	12.07	11.65	8.75	13.86	13.28	12.04	12.04	12.59	–
CA199 (0–27 U/mL)	7.44	–	16.6	17.14	16.9	16.23	18.92	23.37	–	20.43	20.43	16.56	16.18
CA125 (0–35 U/mL)	↑ 154.1	–	16.89	14.07	14.44	16.37	19.39	11.81	13.78	16.01	16.01	13.18	–
CA211 (0–3.3 ng/mL)	↑ 4.48	–	2.23	–	1.58	–	3.18	1.53	1.68	1.87	1.87	1.84	1.53

Dynamic follow-up of tumor markers supported a radiographic complete response (rCR) status. ↑ Indicates that the value is elevated, that is, above the reference range/upper limit of normal.

#### Ultrasonography of the breast and draining lymph nodes:

1) Local thickening and echoic changes (no blood supply) of the glands under the left breast nipple were revealed. An MRI was recommended to exclude a glandular disorder type of breast cancer, BI-RADS 4a. 2) Multiple left axillary lymph nodes were enlarged, and the size of the largest lymph node was approximately 15 * 5 * 12 mm. 3) There was no obvious space-occupying lesion in the right breast.

#### Mammography

The right breast mass was classified as Breast Imaging Reporting and Data System (BI-RADS) category 2, and the left breast mass as BI-RADS category 4.

#### Lung and upper and lower abdominal CT

1) Multiple nodules were found in the left lung, left pleura, mediastinum, and peritoneum, which were considered to be metastases. 2) There was enlargement of the left axillary lymph node. 3) There was a small left pleural effusion. 4) Multiple low-density shadows in the liver were considered to be hepatic cysts. 5) There was mild dilatation of both renal pelves.

#### Breast MRI

1) A mass in the outer and lower quadrant of the left breast was considered to have a high possibility of breast cancer (BI-RADS category 5). 2) There were multiple enlarged lymph nodes in the left axilla. 3) Bilateral breast fibrocystic degeneration (BI-RADS category 3) was found.

Bone scan showed no evidence of metastasis. Puncture pathology of the left breast mass (left breast mass) revealed invasive carcinoma, inclined to invasive ductal carcinoma, Scarff-Bloom-Richardson (SBR) grade I. Immunohistochemistry revealed estrogen receptor (ER) (+++, 90%+), progesterone receptor (PR) (+++, 90%+), HER2 (1+), and Ki-67 (+, positive proportion approximately 5%); original magnification, H&E, ×20 (a–d), 4 × 10 (**Figure 2**).

**Figure 2:**
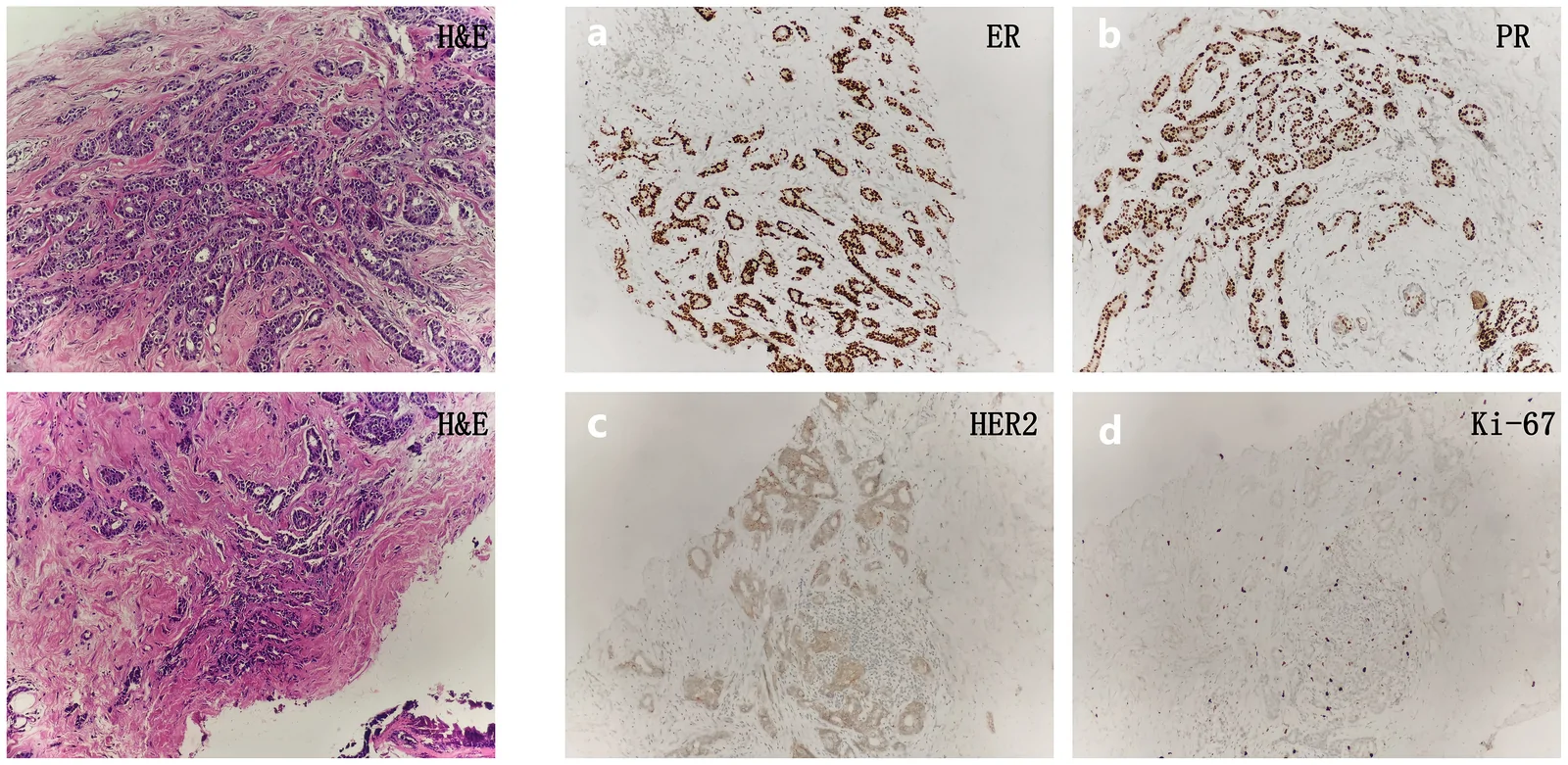
Detailed pathological examination and immunohistochemistry of the patient. Puncture pathology of the left breast mass (left breast mass): invasive carcinoma, inclined to invasive ductal carcinoma, SBR grade I, immunohistochemistry: **(a–d)** ER (+++, 90%+), PR (+++, 90%+), HER2 (1+), Ki-67 (+, positive proportion approximately 5%); original magnification, H&E, ×20 **(a–d)**, 4 × 10.

#### Left axillary lymph node puncture

Left axillary lymph node puncture cytology showed an atypical epithelial cell mass, which was considered to be adenocarcinoma metastasis.

### Therapeutic interventions and follow-up

After pathological confirmation of breast cancer with lung, pleural, mediastinal, and lymph node metastases, the patient was diagnosed with HR-positive, HER2-low invasive ductal breast cancer, stage IV. Per guidelines, she received first-line targeted endocrine therapy with palbociclib + ET. The medication regimen was palbociclib (125 mg PO qd, 3 weeks on medication, 1 week off) + fulvestrant (500 mg IM, administered at 4-week intervals after a loading dose). The patient received 3 cycles (nearly 3 months) of first-line treatment at Shanghai Fudan University Shanghai Cancer Center in December 2019 and returned to our hospital for further therapy in February 2020. After 3 months of combined therapy, partial response was observed in the lung, pleural, mediastinal, and lymph node metastases. Notably, the patient developed symptoms of myelosuppression (leukopenia and neutropenia, **Supplementary Figure 1**) 10 months after receiving treatment, without experiencing other discomforts or adverse effects. Detailed blood routine monitoring changes are available in **Table 2**.

**Table 2:** Blood routine examination.

Examination time	2020-02-26	2020-07-29	2020-09-16	2021-01-26	2021-05-19	2021-08-23	2022-02-18	2022-08-02	2023-08-07	2024-08-21	2024-10-17	2025-05-20	2025-12-17
red lood cell (RBC) (3.8–5.1 * 10^12^/L)	3	2.69	2.95	2.79	2.75	2.44	2.31	2.72	2.72	2.85	3.08	2.89	3.19
white blood cell (WBC) (3.5–9.5 * 10^9^/L)	3.4	8.3	2.4	2.7	1.9	2.3	2.4	2.4	3.3	2.4	2.7	2.6	3
hemoglobin (HB) (115–150 g/L)	109	105	113	114	106	102	93	115	114	110	117	113	124
platelet (PLT) (125–350 * 10^9^/L)	109	143	103	139	100	75	78	161	156	116	92	97	115
neutrophil (NEU) (1.8–6.3 * 10^9^/L)	1.2	4.9	0.8	0.7	0.7	0.7	0.9	0.6	0.8	0.7	1.4	0.6	1.1

The results showed that the patient suffered adverse reactions of granulocytopenia and leukopenia following the initiation of first-line treatment therapy in the 10th month.

At 18 months, repeat enhanced CT scans revealed a radiographic complete response (rCR). Up to now, the patient has maintained radiographic remission for more than 6 years without complaints and side effects other than neutropenia and leukopenia (sustained rCR). Comparative imaging examinations during treatment are shown in **Figure 3**. Because the case was a glandular disorder type of breast cancer, no exact changes in the size of the breast cancer lesions were recorded during the follow-up diagnosis and treatment process. The key time nodes and events are as follows (**Figure 4**):

**Figure 3:**
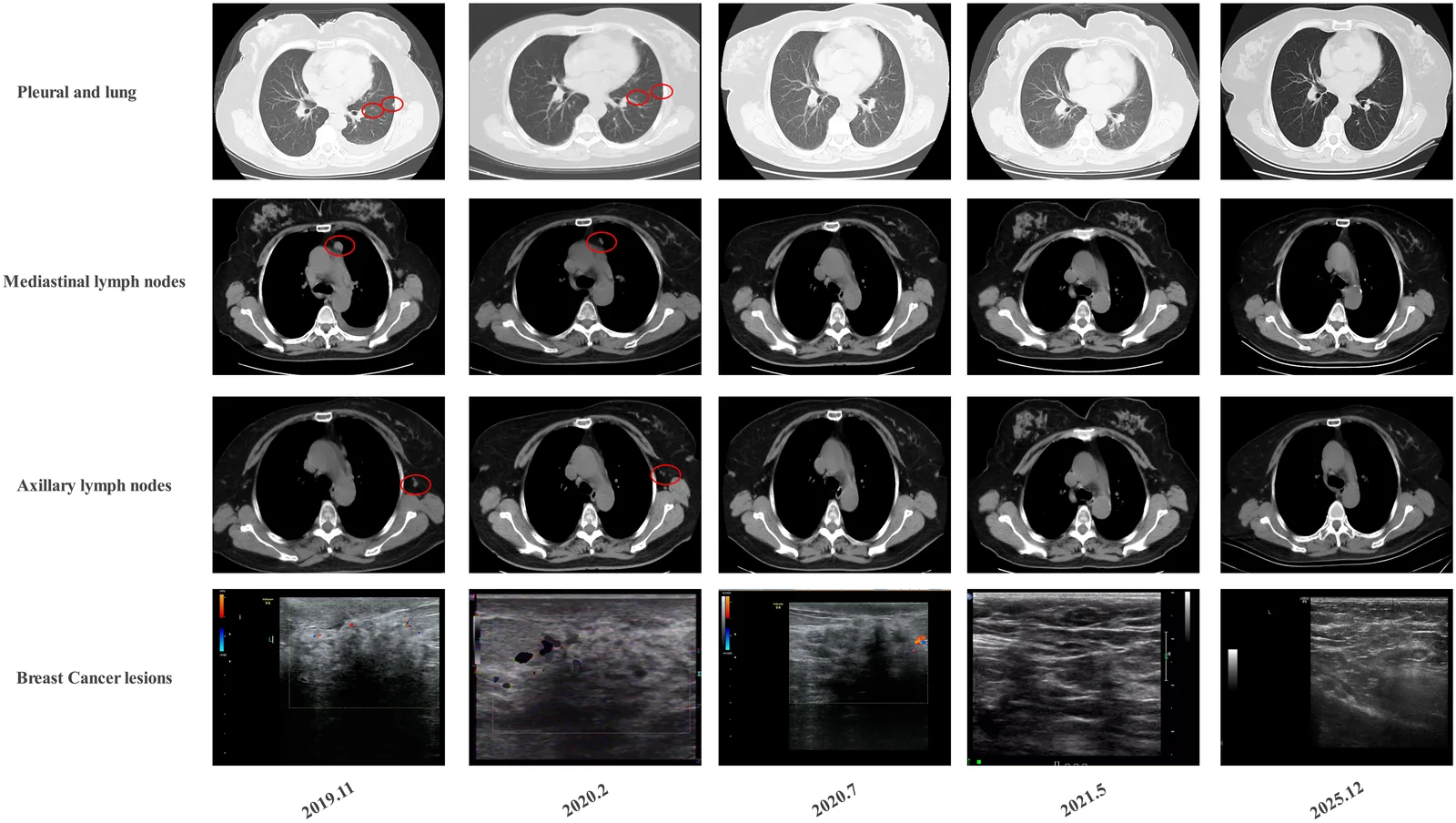
Imaging examination showing complete disappearance of the breast cancer metastases.

**Figure 4:**
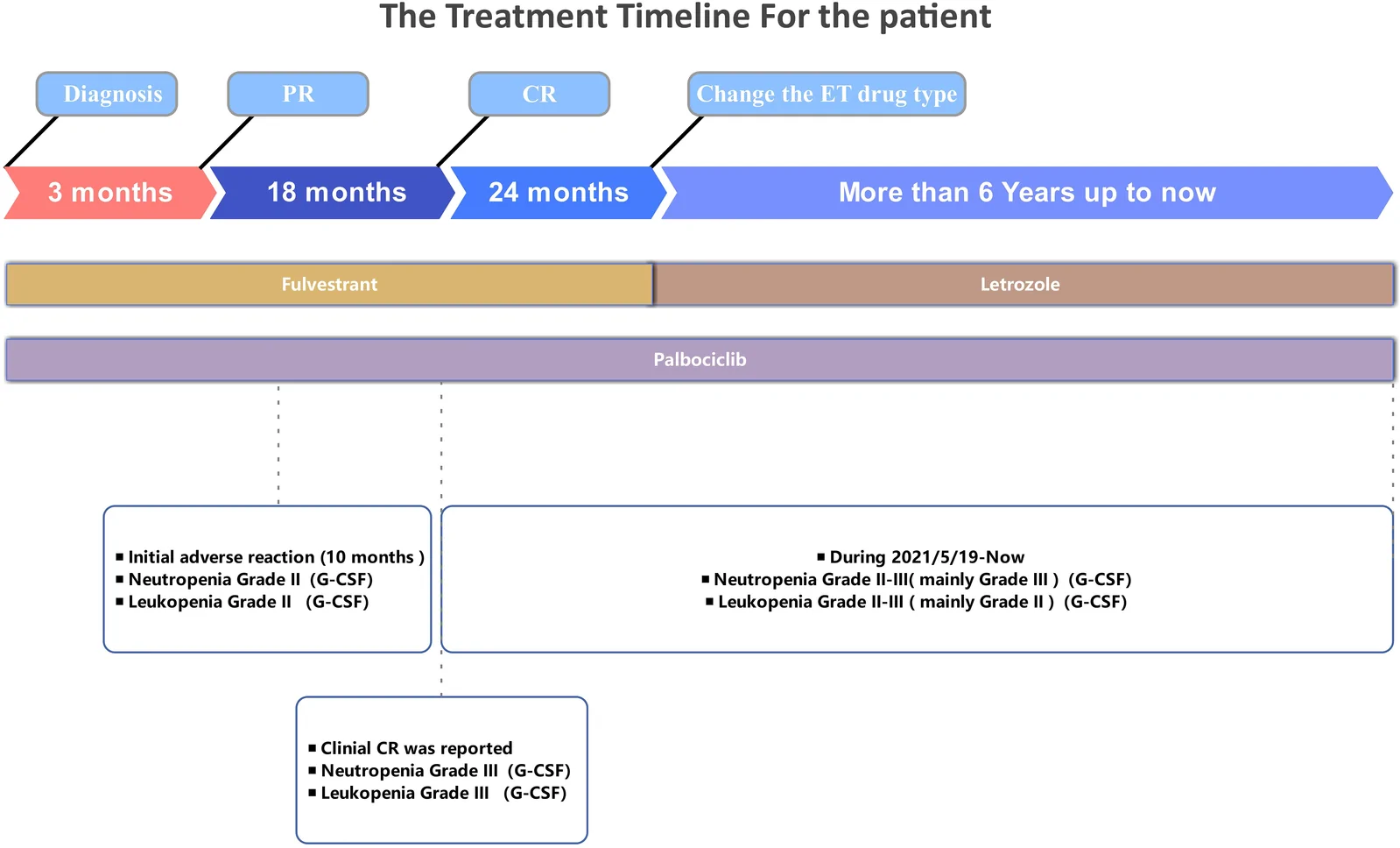
The treatment timeline for the patient. PR, partial response; CR, complete response (radiographic complete response).

November 2019: initial diagnosis, imaging suggested metastasis, and pathological diagnosis

December 2019: started palbociclib + fulvestrant

March 2020: PR

June 2021: rCR

December 2021: fulvestrant changed to letrozole because of injection intolerance

Remission maintained to the latest follow-up

## Discussion

This case report describes a remarkable and sustained radiographic complete response to palbociclib plus fulvestrant, followed by letrozole, in a patient with HR-positive, HER2-low MBC involving the lung, pleura, and lymph nodes. The patient achieved near-complete regression of metastatic lesions after 18 months of therapy and maintained remission for over 6 years despite switching ET due to injection intolerance.

The exceptional and durable complete radiographic response observed in this patient with HR-positive, HER2-low MBC treated with palbociclib-based regimens was a rare clinical event. While CDK4/6 inhibitors combined with ET have significantly improved PFS in HR-positive, HER2-negative MBC, achieving a radiographic CR is uncommon (14). In European and American populations, real-world studies of palbociclib plus letrozole for MBC patients have reported median PFS ranging from 16.5 to 22.2 months, with objective response rates (ORRs) below 30% (15, 16). The same treatment may be more beneficial in the Asian population. The Japanese P-BRIDGE study was a multicenter, observational study involving 693 Japanese patients with HR-positive, HER2-negative ABC who received either first-line (1L; n = 426) or second-line (2L; n = 267) palbociclib plus ET. In the latest P-BRIDGE study (17), the follow-up time was extended to 48.1 months (previously, the median follow-up was only 36 months), and the real-world progression-free survival (rwPFS) was 26.2 months (95% CI 21.4–30.4) in the 1L group and 14.9 months (95% CI 11.7–18.3) in the 2L group. The median OS of the 1L group was 68.2 months [95% CI 60.8 to not reached (NR)], which was better than 50.7 months (95% CI 42.2–57.2) in the 2L group. The OS of patients with visceral metastasis was higher than that of patients with liver and bone metastasis. The sustained rCR exceeding 6 years in this case far surpasses these typical outcomes, aligning also more closely with the prolonged median PFS of 31.8 months reported for palbociclib plus fulvestrant in the endocrine-sensitive FLIPPER trial, although a CR was not specified (18). This case is consistent with current guideline recommendations supporting CDK4/6 inhibitor-based endocrine therapy as standard first-line treatment for HR-positive, HER2-low metastatic breast cancer, especially in Asian populations.

Notably, the patient in this case maintained efficacy following a switch from fulvestrant to letrozole due to injection intolerance. Most fulvestrant is administered via intramuscular injection in the gluteal region. However, this is difficult for patients to tolerate during long-term treatment, especially those with poor baseline conditions. This situation usually receives little attention in clinical trials, but it does lead to patients switching drugs in the absence of resistance to endocrine therapy, which could impact the final research conclusions. Previous reports support the finding that sequential ET with a CDK4/6 inhibitor can be effective (19). In this patient, durable disease control was maintained after switching from fulvestrant to letrozole, suggesting that endocrine partner substitution may be feasible in selected patients when treatment intolerance occurs; however, this observation requires validation in larger studies. This contrasts with cases of acquired resistance, often driven by genomic alterations such as ESR1 mutations or RB1 loss, which typically lead to progression within a shorter timeframe (20). Furthermore, while HER2-low status has been investigated as a prognostic factor, meta-analyses have not consistently shown it to significantly impact PFS on CDK4/6i therapy compared to HER2-zero disease, suggesting that the remarkable response here might not be primarily attributable to the HER2-low phenotype (10). Recent prospective clinical trial evidence suggests that for patients with HR-positive, HER2-positive advanced breast cancer, the addition of palbociclib to maintenance anti-HER2 and endocrine therapies significantly improved progression-free survival compared to standard therapy (21).

Several clinical and pathological features may have jointly contributed to the exceptionally durable response observed in this patient. The tumor showed strong ER and PR expression (both approximately 90%) and a very low Ki-67 proliferation index (approximately 5%). The low Ki-67 index may reflect relatively indolent tumor biology, whereas the strong hormone receptor expression and treatment-naive presentation suggest preserved endocrine dependence. Retrospective studies have associated lower Ki-67 and higher PR expression with longer progression-free survival during palbociclib-based endocrine therapy (22, 23). Because estrogen-driven proliferation is mediated in part through the ER–cyclin D–CDK4/6–RB pathway, the simultaneous inhibition of estrogen signaling and CDK4/6 activity may have produced sustained cell-cycle arrest (24). Moreover, comparative biomarker analyses of the PALOMA-2 and PALOMA-3 trials found that sensitivity-associated genes were enriched in estrogen response pathways, further supporting a potential relationship between endocrine-responsive tumor biology and palbociclib benefit (25). Endocrine therapy naivety and very high ER expression have also been associated with prolonged survival in patients treated with CDK4/6 inhibitors, while the absence of liver metastasis has been associated with more favorable survival in univariate analysis (26).

Beyond cytostasis, CDK4/6 inhibition may induce cell enlargement and senescence; immune-mediated clearance of senescent tumor cells could plausibly contribute to tumor regression (27). These may be related to the good efficacy in this patient.

Regrettably, due to economic pressure and personal will, although the patient still adhered to the treatment, she did not regularly undergo pathological and bone metabolism PET–CT examinations after achieving rCR, and thus, we could not confirm whether she had achieved pathological CR. Furthermore, further studies are needed to elucidate the molecular and biological basis of such exceptional responses. The absence of molecular profiling represents an important limitation, and whether this patient reflects a biologically distinct subgroup remains uncertain. Overall, this case provides valuable information for the future application of palbociclib in the treatment of patients with HR-positive, HER2-negative advanced breast cancer.

## Conclusion

This case illustrates that palbociclib combined with endocrine therapy can induce a durable radiographic remission in selected patients with HR-positive, HER2-low metastatic breast cancer. In patients who develop intolerance to one endocrine partner, switching to an alternative endocrine agent while maintaining CDK4/6 inhibition may be feasible, but this observation requires validation in larger studies.

## Data Availability

The original contributions presented in the study are included in the article/**Supplementary Material**. Further inquiries can be directed to the corresponding author.

## Supplementary material

Supplementary Figure 1 Line graph monitoring the trend in white blood cell and neutrophil counts.
